# Efficacy and safety of combined endoscopic cyanoacrylate injection and balloon-occluded retrograde transvenous occlusion (BRTOcc) of gastrorenal shunts in patients with bleeding gastric fundal varices

**DOI:** 10.1093/gastro/goaa082

**Published:** 2020-12-03

**Authors:** Fateh Bazerbachi, Akira Dobashi, Swarup Kumar, Sanjay Misra, Navtej S Buttar, Louis M Wong Kee Song

**Affiliations:** 1 Division of Gastroenterology, Interventional Endoscopy Program, Massachusetts General Hospital and Harvard Medical School, Boston, MA, USA; 2 Division of Gastroenterology and Hepatology, Mayo Clinic, Rochester, MN, USA; 3 Vascular and Interventional Radiology, Mayo Clinic, Rochester, MN, USA

**Keywords:** balloon-occluded retrograde transvenous occlusion, cyanoacrylate injection, gastric varices, gastric variceal bleeding, gastrorenal shunt

## Abstract

**Background:**

Endoscopic cyanoacrylate (glue) injection of fundal varices may result in life-threatening embolic adverse events through spontaneous gastrorenal shunts (GRSs). Balloon-occluded retrograde transvenous occlusion (BRTOcc) of GRSs during cyanoacrylate injection may prevent serious systemic glue embolization through the shunt. This study aimed to evaluate the efficacy and safety of a combined endoscopic–interventional radiologic (BRTOcc) approach for the treatment of bleeding fundal varices.

**Methods:**

We retrospectively analysed the data of patients who underwent the combined procedure for acutely bleeding fundal varices between January 2010 and April 2018. Data were extracted for patient demographics, clinical and endoscopic findings, technical details, and adverse events of the endoscopic–BRTOcc approach and patient outcomes.

**Results:**

We identified 30 patients (13 [43.3%] women; median age 58 [range, 25–92] years) with gastroesophageal varices type 2 (53.3%, 16/30) and isolated gastric varices type 1 (46.7%, 14/30) per Sarin classification, and median clinical and endoscopic follow-up of 151 (range, 4–2,513) days and 98 (range, 3–2,373) days, respectively. The median volume of octyl-cyanoacrylate: Lipiodol injected was 7 (range, 4–22) mL. Procedure-related adverse events occurred in three (10.0%) patients, including transient fever, non-life-threatening pulmonary glue embolism, and an injection-site ulcer bleed. Complete gastric variceal obturation was achieved in 18 of 21 patients (85.7%) at endoscopic follow-up. Delayed variceal rebleeding was confirmed in one patient (3.3%) and suspected in two patients (6.7%). Although no procedure-related deaths occurred, the overall mortality rate was 46.7%, primarily from liver-disease progression and co-morbidities.

**Conclusion:**

The combined endoscopic–BRTOcc procedure is a relatively safe and effective technique for bleeding fundal varices, with a high rate of variceal obturation and a low rate of serious adverse events.

## Introduction

Gastric varices (GV) are an important cause of upper gastrointestinal bleeding and are found in 20% of patients with portal hypertension [1]. Although GV tend to bleed less frequently than esophageal varices, bleeding is more severe and is associated with higher mortality.

Sarin classification is used to categorize GV, based on anatomic location and relationship with esophageal varices. Gastroesophageal varices (GOV) are GV that occur in the presence of esophageal varices; GOV1 are an extension of esophageal varices along the cardia and gastric lesser curvature (cardia varices), whereas GOV2 are gastroesophageal varices that extend toward the fundus. Isolated gastric varices (IGV) occur in the absence of esophageal varices and are subclassified as IGV1 when the varices are located in the fundus or IGV2 when the varices are present at other locations in the stomach (e.g. the antrum).

Regarding the endoscopic treatment of bleeding GV, the management of GOV1 is similar to that of esophageal varices, whereas cyanoacrylate (glue) injection is the preferred therapy for fundal varices (GOV2 and IGV1) [2]. Although limited data exist on the optimal treatment of IGV2, the latter is usually managed in a similar fashion to fundal varices.

Following the initial description of cyanoacrylate injection for the treatment of GV by Soehendra *et al*. [3] in 1986, subsequent studies have demonstrated excellent hemostasis rates using this technique [4–6]. Cyanoacrylate injection, however, requires careful patient selection and attention to technique, since the majority of patients with fundal varices harbor spontaneous portosystemic shunts, in particular gastro- or splenorenal shunts (GRSs), which are the main conduits for potentially life-threatening systemic glue-embolization events.

Techniques aimed at minimizing the risk of systemic glue embolization are of interest to improve procedural safety. One such technique is the balloon-occluded retrograde transvenous obliteration (BRTO) of portosystemic shunts. This interventional radiologic (IR) technique requires the presence of a GRS and was introduced in the 1990s as a stand-alone method for the treatment of GV [7]. It involves the temporary occlusion of the GRS using a balloon-occlusion catheter inserted via a transjugular or transfemoral route, followed by the instillation of a sclerosing agent or foam into the GV [7]. Although effective, BRTO has been associated with a host of adverse events, including arrhythmias, intravascular hemolysis, disseminated intravascular coagulation, renal injury, and the development or worsening of esophageal varices [8]. Moreover, the dwell time for the balloon-occlusion catheter ranges from 4 to 48 hours, which can be associated with infection, adverse events at the access site, and intravascular balloon rupture [9].

**Figure 1.**
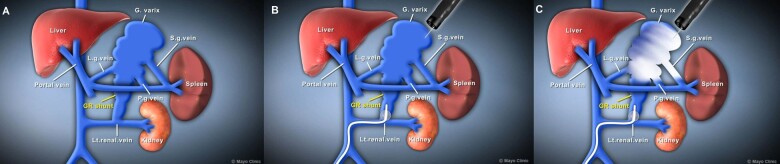
Schematic representation of combined endoscopic cyanoacrylate injection and balloon-occluded retrograde transvenous occlusion (BRTOcc) of gastrorenal shunts in patients with bleeding gastric fundal varices. (A) Relationship of the gastrorenal shunt (GRS) relative to the gastric varices and systemic circulation (left renal vein); (B) angiographic catheter balloon occlusion of the GRS via the transfemoral route; (C) endoscopic injection of cyanoacrylate-fluoroscopic contrast mixture into the gastric varices during balloon occlusion of the GRS. G varix, gastric varix; GR shunt, gastrorenal shunt; L.g. vein, left gastric vein; Lt.renal.vein, left renal vein; P.g.vein, posterior gastric vein; S.g.vein, short gastric vein.

Although cyanoacrylate is a more effective obturating agent for GV than a sclerosant or foam, the injection of cyanoacrylate through the balloon-occlusion catheter during BRTO is ill-advised due to the rapid polymerization of the glue and risk of cementing the catheter within the GRS. However, the concept of occluding the GRS during endoscopic cyanoacrylate injection is appealing, since it provides a means of preventing potentially serious glue embolization through this major portosystemic shunt (Figure 1). Moreover, the dwell time for balloon occlusion of the GRS following glue injection is significantly shorter, in the order of minutes as opposed to hours, for BRTO. Imazu *et al*. [10] initially described the technique of endoscopic cyanoacrylate injection following angiographic balloon occlusion of GRSs in two patients, with successful obliteration of the varices. Our group published a similar procedure for the treatment of bleeding fundal varices in 2012 [11]. Subsequently, this technique has been applied by other groups and reported as small case series with limited follow-up [12, 13].

Herein, we describe our experience regarding the combined endoscopic–IR approach for the treatment of fundal variceal hemorrhage utilizing endoscopic cyanoacrylate injection with balloon-occluded retrograde transvenous occlusion (BRTOcc) of the GRS.

## Patients and methods

### Study design

We reviewed the electronic medical records of patients who underwent combined endoscopic cyanoacrylate injection with BRTOcc of GRSs for bleeding fundal varices from January 2010 to April 2018 at Mayo Clinic, Rochester, Minnesota, USA. All patients included in the study had authorized the use of their medical records for research review and the study was carried out following approval from the Mayo Clinic Institutional Review Board (IRB#14–004811). The investigation conforms with the principles outlined in the Declaration of Helsinki.

### Patients

Patients presenting with acute upper-GI bleeding and the presence of fundal varices (GOV2 or IGV1) with active or definite stigmata of recent bleeding (e.g. adherent clot, fibrin plug) on initial endoscopy were considered for the combined procedure. If active bleeding was encountered at the time of the initial endoscopy, temporary control of bleeding was achieved either with band ligation or through-the-scope clip placement. Pre-procedure assessment included cross-sectional imaging (computed tomography or magnetic resonance imaging) to document the presence of a GRS and contrast echocardiography to determine the presence of intracardiac shunts. In the absence of a GRS, straight endoscopic undiluted cyanoacrylate injection was offered as a treatment option. In the presence of an intracardiac shunt, alternative treatment options were favored, including transjugular intra-hepatic portosystemic shunt (TIPS) and endoscopic ultrasound (EUS)-guided coil injection. Informed consent was obtained from the patient or his/her surrogate after discussing the risks and benefits of cyanoacrylate injection as an off-label therapy for GV.

### Technique

**Figure 2.**
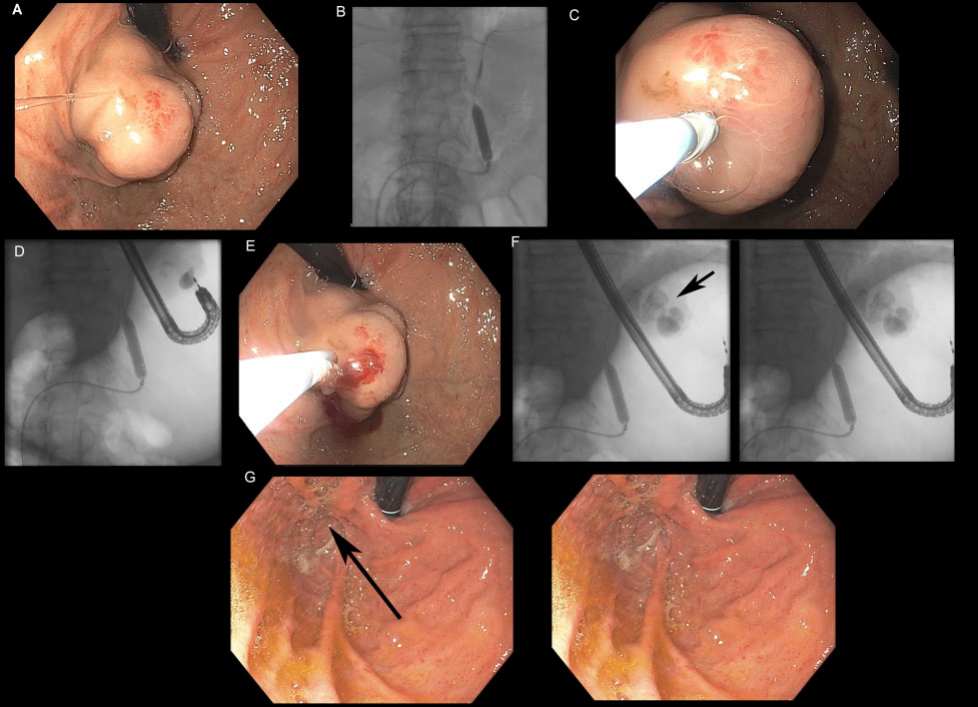
A case of combined endoscopic–BRTOcc therapy. (A) Large isolated gastric varices type 1; (B) balloon-occluded retrograde transvenous occlusion (BRTOcc) of gastrorenal shunt; (C) endoscopic injection of cyanoacrylate–Lipiodol mixture; (D) real-time fluoroscopic monitoring of injection therapy with BRTOcc; (E) completion of endoscopic injection therapy; (F) complete solidification and obturation of the injected fundal varices; (G) complete fundal variceal obliteration on endoscopic follow-up at 7 months.

The endoscopic–BRTOcc procedure was performed in the interventional radiology suite, with the patient in a supine position and under general anesthesia. Pre-procedure antibiotic prophylaxis was administered. At the discretion of the interventional radiologist and based on vascular anatomy, either a transfemoral or a transjugular approach was used to position an angiographic balloon-occluding catheter (e.g. Opta Pro PTA, Cordis, Santa Clara, CA, USA) over a guide wire through the GRS. Immediately following balloon occlusion of the GRS, a standard adult endoscope was introduced into the stomach and the GV was injected with a mixture of 2-octyl-cyanoacrylate (Dermabond^®^, Ethicon, Somerville, NJ, USA) and a radiopaque contrast agent (Lipiodol^®^, Guerbet, Princeton, NJ, USA). The Dermabond–Lipiodol mixture was injected via 3-mL syringes, in a 2.5:0.5 mL ratio, using a dedicated injection needle (Marcon-Haber Varices Injector, Cook Medical, Bloomington, IN, USA). Typically, only one needle puncture was performed adjacent to the bleeding stigmata and the injectate was monitored fluoroscopically, with the endpoint being complete solidification of the variceal complex by the glue-fluoroscopic contrast material (Figure 2). The balloon-occluding catheter was deflated and withdrawn 10 min after endoscopic injection. Follow-up endoscopy was performed at 1 month to assess treatment response and confirm the obliteration of the GV, with or without the use of an endoscopic Doppler probe (Vascular Technology, Inc. [VTI], Nashua, NH, USA) and every 6 months thereafter to monitor for recurrence.

### Data collection and analysis

Data were abstracted for patient demographics, presenting symptoms, and clinical history that included underlying liver disease and co-morbidities, laboratory results, the model for end-stage liver disease (MELD) score, endoscopic findings, type of GV and bleeding stigmata, technical details of the endoscopic–BRTOcc procedure and related adverse events, and post-treatment outcomes.

Statistical analysis was performed utilizing the SPSS software version 25 (IBM Corp. Released 2017. IBM SPSS Statistics for Windows, Version 25.0. Armonk, NY: IBM Corp, USA), including univariate and multivariate analysis of patient and endoscopic characteristics to predict the outcomes of the procedure. Procedure-related minor and major adverse event rates, as well as rebleeding rates, were estimated for the study group. A *P*-value of <0.05 was considered statistically significant.

### Case-series risk of bias

We relied on a newly published tool to provide a quality assessment of the risk of bias in our reported case series [14]. This tool has been widely used and applied in previous publications [15–24]. All patients represented the whole experience of our center during the study period. The exposure (endoscopic–BRTOcc) was ascertained for all cases. The outcome (bleeding) was adequately ascertained in all cases. No other alternative causes explained the outcome of bleeding. Follow-up was adequate for the assessment of the outcome. The methodology of all cases was identical, allowing other investigators to emulate the intervention.

## Results

### Baseline characteristics

During the study period, 30 patients underwent the combined endoscopic–IR procedure (Table 1). All patients were hospitalized with acute upper-GI bleeding, with presenting symptoms of hematemesis in 12 (40.0%), melena in 12 (40.0%), and/or hematochezia in 8 (26.7%). Thirteen (43.3%) patients required packed red blood cell transfusions.

**Table 1.** Clinical, endoscopic, and treatment outcomes in 30 patients who underwent the combined endoscopic–interventional radiologic procedure

Characteristic	Value
Patients, *n*	30
Female gender, *n* (%)	13 (43.3%)
Age, median (range), years	58 (25–92)
Hemoglobin on admission, median (range), g/dL	10.4 (4–14.4)
INR on admission	1.35 (0.9–2.3)
Na-MELD score on admission	14 (7–27)
Prior TIPS placement, *n* (%)	4 (13.3%)
Type of gastric varices: IGV1/GOV2	46.7%/53.3%
Active variceal bleeding at index endoscopy, *n* (%)	4 (13.3%)
Volume of octyl-cyanoacrylate: Lipiodol mixture injected, median (range), mL	7 (4–22)
Procedure-related adverse events, *n* (%)	3 (10.0%)
Rebleeding GV (confirmed), *n* (%)	1, (3%)
Rebleeding GV (suspected), *n* (%)	2, (6.7%)
Clinical follow-up, median (range), days	151 (4–2,513)
Endoscopic follow-up, median (range), days	98 (3–2,373)
Lost to endoscopic follow-up, *n* (%)	9 (30%)
Gastric variceal obliteration at follow-up endoscopy, *n* (%)	18/21 (85.7%)
GV persistence/recurrence at follow-up endoscopy, *n* (%)	3/21 (14.3%)

GV, gastric varices; GOV, gastroesophageal varices; IGV, isolated gastric varices; INR, international normalized ratio; mL, milliliter; MELD, model for end-stage liver disease; Na, sodium; TIPS, transjugular intra-hepatic portosystemic shunt.

### Endoscopic features

All patients underwent endoscopy within 24 h of presentation, with identification of GV as the bleeding source. All fundal varices were large (>5 mm) and classified as IGV1 (*n *=* *14, 46.7%) or GOV2 (*n *=* *16, 53.3%). Four patients (13.3%) had active bleeding at the time of endoscopy. Initial hemostasis of active bleeding was achieved through balloon tamponade in one patient, band ligation in one patient, and hemoclip placement in two patients. Definitive therapy with endoscopic–BRTOcc was accomplished within 48 h of presentation in all patients.

### Portal hypertension features

Four patients (13.3%) had prior TIPS placement, with two (6.7%) having failed TIPS for bleeding control prior to being considered for the combined therapy. The etiologies of portal hypertension varied, with the majority (56.7%, 17/30) being due to alcoholic and/or hepatitis C cirrhosis. The median (range) Na-MELD score was 14 (7–27) and the Child–Turcotte–Pugh (CTP) score was 8 (5–12).

### Endoscopic–BRTOcc features

Cross-sectional imaging was falsely positive for the presence of a GRS in one patient and therefore the angiographic balloon was not inflated. Otherwise, the endoscopic–BRTOcc procedure was technically successful in all patients. Angiographic access to the GRS was obtained via the transfemoral route in 26 (86.7%) patients and the transjugular route in 4 (13.3%) patients. The median (range) injected volume of the cyanoacrylate–Lipiodol mixture was 7 mL (4–22 mL). In two-thirds of procedures, a single injection site was performed. Two separate injection sites were performed for the remaining third. Filling and solidification of the variceal complex at fluoroscopy and hardening of the GV upon palpation with the injection catheter at endoscopy constituted the technical endpoints of treatment.

### Treatment-adverse events

Three patients (10.0%) developed procedure-related adverse events, including transient fever, post-treatment bleeding fundal ulcer successfully treated with additional cyanoacrylate injection, and pulmonary glue embolism. Post-procedure pleuritic chest pain in the latter patient prompted a chest computed tomography, which confirmed bilateral tiny subpleural glue emboli and which resolved with conservative management. The delay between initial endoscopy and subsequent combined IR–endoscopy for glue injection did not result in adverse events. Patients who had active bleeding at the time of initial endoscopy were temporized with measures (e.g. clip placement) until the combined procedure.

### Follow-up outcomes

Twenty-one patients (70.0%) had at least one endoscopic follow-up and 85.7% (18/21) of those patients demonstrated complete GV obliteration. One patient had delayed GV bleeding at 3 months after incomplete GV eradication. Regression analysis did not identify a statistically significant factor that relates to successful endoscopic GV obliteration. However, overall mortality was associated with the need for blood transfusion (*P *=* *0.01), increased number of transfused units (*P *=* *0.04), presence of encephalopathy (*P *=* *0.05), and worse CTP score (*P *=* *0.01), but not worse Na-MELD score.

**Figure 3.**
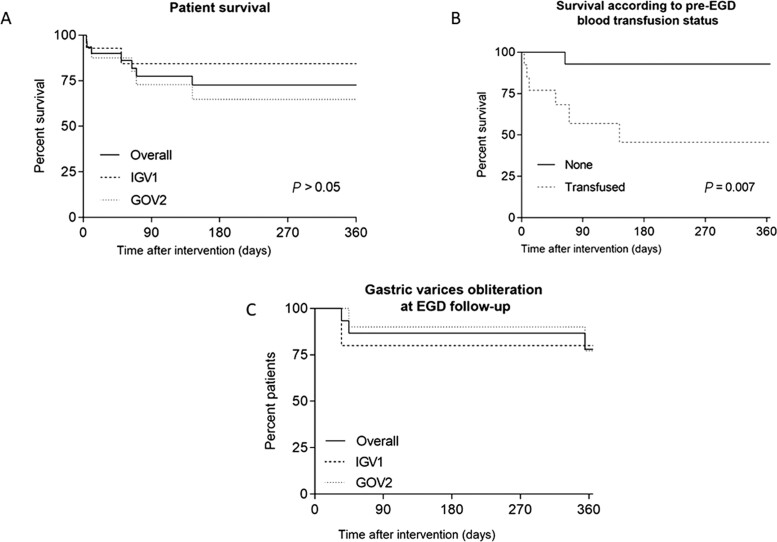
Kaplan–Meier curves survival analyses figures. (A) Patient survival according to types of GV; (B) patient survival according to pre-endoscopic blood-transfusion status; and (C) gastric variceal obliteration status post endoscopic–BRTOcc approach for bleeding fundal varices on follow-up endoscopy.

The median (range) clinical follow-up period was 151 (4–2,513) days and endoscopic follow-up was 98 (3–2,373) days to determine persistent obliteration of the varices. Figure 3 shows Kaplan–Meier survival curves for patients according to GV subtype (A), pre-endoscopic–BRTOcc transfusion status (B), and GV-recurrence-free survival according to endoscopic follow-up and GV subtype (C).

### Mortality

The overall death rate for the patient cohort was 47% (*n *=* *14). Among patients who died during follow-up, two patients succumbed within 2 months of the procedure secondary to presumed recurrent GV bleeding, although this was not confirmed by endoscopy. There were no deaths attributed directly to the procedure and the overall death rate was related to the patients’ underlying chronic illnesses and/or natural history of their underlying liver disease.

### Risk of bias

Our case series showed a low risk of bias in totality.

## Discussion

Bleeding from fundal varices can be severe and is associated with higher mortality than esophageal variceal hemorrhage [2]. Cyanoacrylate injection is considered first-line endoscopic therapy for fundal variceal hemorrhage [4] and is more effective than sclerotherapy and band ligation [25]. EUS-guided angiotherapy with coil and/or glue injection is a promising alternative, but this technique requires an experienced endosonographer and may not be readily available in the acute setting [26].

From an IR perspective, BRTO is an effective treatment modality for bleeding GV. Compared with cyanoacrylate injection, BRTO is associated with lower odds of rebleeding and higher odds of complete GV obliteration according to a recent meta-analysis [27]. However, the large volume of instilled sclerosant during BRTO dissipates in the systemic circulation, potentially leading to profound systemic disturbances and deleterious adverse events. In this regard, endoscopic cyanoacrylate injection is appealing because the volume injected is relatively small and its effect consists primarily of localized vascular thrombosis. Technical variations on BRTO have been proposed. Coil-assisted retrograde transvenous obliteration (CARTO) is one such treatment option for bleeding GV, but it may lead to worsening of ascites and/or hydrothorax [28]. None of our patients suffered from such complications. Our combined technique entails a single percutaneous access route, obviating the need for a second percutaneous site as is the case with balloon-assisted percutaneous transhepatic antegrade embolization, another IR variant [29]. Although TIPS can be successful at managing bleeding GV, it is associated with a worse complication profile than BRTO [30]. Unlike TIPS, which may initiate or worsens encephalopathy, BRTO or the endoscopic–BRTOcc procedure may be a viable option in patients who are at high risk for encephalopathy. Furthermore, many patients with GV harbor underlying portal vein thrombosis, which may impede TIPS placement.

The technique of endoscopic cyanoacrylate injection is not standardized and, often, only a small amount of glue (2–3 mL) is injected in the hope of obtaining adequate variceal obturation while minimizing the risk of glue embolization. However, even a small amount of glue injected can result in systemic embolization, as evidenced by the presence of pulmonary glue embolism on chest computed tomography in as many as 47% of patients who underwent such a procedure, albeit with the majority of these patients remaining asymptomatic [31]. Although the risk of fatal glue embolism appears low, it is increased in the setting of large GV that requires a larger amount of glue and in patients who harbor hemodynamically significant GRS. We surmise that serious, potentially fatal embolic complications may be mitigated by occluding the GRS during endoscopic cyanoacrylate injection. Moreover, in our study, it became apparent that a larger volume of cyanoacrylate was required to obtain satisfactory obturation of the variceal complex when performed with real-time fluoroscopy.

Our combined technique, therefore, capitalizes on the protection from major embolic events by angiographic occlusion of the GRS (BRTOcc) while endoscopically injecting the required amount of cyanoacrylate needed under fluoroscopic monitoring to obtain complete vascular obturation of the GV. Moreover, balloon occlusion of the GRS causes relative stagnation of blood flow within the GV, enabling more efficient contact and polymerization of the glue in the variceal complex. In most cases, only one endoscopic needle puncture is needed to achieve satisfactory vascular obturation, as evidenced by real-time fluoroscopy. Unlike stand-alone BRTO, the balloon-occluding catheter can be removed within minutes, following endoscopic glue injection. Of note, however, one patient in the study suffered from a nonfatal pulmonary glue embolism. While the combined endoscopic–BRTOcc technique minimizes the risk of clinically serious, glue-related adverse events through the GRS, it does not eliminate the incidence of subclinical or mildly symptomatic small-particle glue embolization through other smaller portosystemic collaterals.

In our patient cohort, the endoscopic–BRTOcc procedure was associated with a relatively low rebleeding rate and a low GV-recurrence rate in those who received follow-up endoscopy. The technique did not worsen pre-existing encephalopathy and was applied successfully in the setting of portal- and splenic-vein thromboses. Furthermore, the procedure was successful in four patients with bleeding GV despite patent TIPS, without recurrence of GV at endoscopic follow-up. The potential utility of the combined procedure in high-risk patients with bleeding GV who are not candidates for or have failed TIPS placement should not be underestimated. Although mortality neared 50% on long-term follow-up (up to 8 years) in our patient cohort, this mortality rate is a reflection of the natural history of the underlying liver disease and co-morbidities. Indeed, the 6-month survival was ∼80%, which is a suitable endpoint for an emergent life-saving procedure.

Our study has several inherent limitations. First, our series is limited by the retrospective-study design and the relatively small sample size. In particular, we cannot exclude a type II error and differences in survival that may become apparent with larger sample sizes. Second, endoscopic follow-up was not available in all patients. Third, our series excluded IGV2 patients, thus limiting the generalizability of our findings regarding the treatment of varices at other locations in the stomach. On the other hand, the procedural steps were standardized and, to our knowledge, this study represents the largest single-center series with the longest follow-up to date reporting on the combined endoscopic–IR procedure for bleeding fundal varices.

In conclusion, the combined endoscopic–BRTOcc approach appears to be efficacious and safe for the management of bleeding fundal varices in patients with GRS. Further studies addressing this technique on larger sample sizes and in comparison with existing modalities, including TIPS and BRTO, are awaited.

## Authors’ contributions

All authors contributed to the study conception and design. F.B. and A.D. performed material preparation, data collection, and analysis. F.B. and L.M.W.K.S. drafted the manuscript, and all authors commented on previous versions of the manuscript. All authors read and approved the final manuscript.

## Funding

None.

## Conflicts of interest

None declared.
